# Ectopic pancreatic tissue in a cholecystectomy specimen: A rare incidental pathologic finding

**DOI:** 10.1002/ccr3.7961

**Published:** 2023-10-06

**Authors:** Nooshin Zaresharifi, Anita Khalili, Behrad Eftekhari, Hojjat Layegh

**Affiliations:** ^1^ Department of Pathology Guilan University of Medical Sciences (GUMS) Rasht Iran; ^2^ Department of Medicine Guilan University of Medical Sciences (GUMS) Rasht Iran; ^3^ Department of Plastic Surgery, Panzdahe Khordad Hospital, School of Medicine Shahid Beheshti University of Medical Sciences Tehran Iran

**Keywords:** case report, cholecystitis, ectopic pancreas, gallbladder

## Abstract

Ectopic pancreatic tissue is a rare congenital abnormality defined as the abnormal location of pancreatic tissue outside the anatomical site of the pancreas without any anatomical or vascular connection to it, which is often discovered incidentally. This is a case of a 40‐year‐old man who was admitted to our surgical department for emergency cholecystectomy due to acute gangrenous cholecystitis. Preoperative ultrasound imaging was indicative only of multiple stones in the gallbladder. Postoperative histopathological examination revealed an area of wall thickening in the neck region of the gallbladder consists of ectopic pancreatic tissue. We emphasize the role of a precise pathologic examination even in routine surgical specimens such as a cholecystectomy specimen, since no preoperative evaluation can be affirmative of such incidental but momentous histopathologic findings. Anatomical pathologists must be aware of the rare presentation of Ectopic Pancreatic Tissue (EPT) in gallbladder which may masquerade as a malignancy.

## Graphical abstract

Below is depicted histomorphology of gallbladder wall *asterisks* with an incidental erratic occurrence of heterotopic pancreatic islands *arrows and arrow heads* in an acute gangrenous cholecystectomy specimen *right upper corner*, masquerading malignancy in the initial sections of microscopic examination.

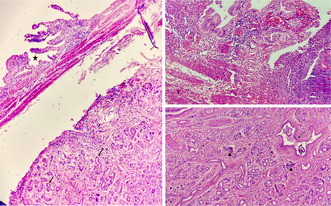

## 1 INTRODUCTION

Ectopic pancreatic tissue (EPT) or pancreatic heterotopia is a rarely observed congenital abnormality defined as the presence of pancreatic tissue in another organ without any anatomical or vascular connection to the pancreas. The term consists of the two Greek words “hetero‐” which means “other” and “‐topia” which means “site,” pointing out the unique location of pancreatic cells. EPT's favored sites are the stomach, duodenum, colon, jejunum, and Meckel's diverticula.^1^ The gallbladder is a highly infrequent location for EPT.^2^ Almost all cases are detected incidentally during the histopathological examination after cholecystectomy for other pathologies. The prevalence of EPT in the gastrointestinal tract varies from 0.6% to 13.7% in autopsy series and 0.2% in laparotomies.^3^, ^4^ Although the malignant transformation of this tissue is not frequently expected, pathologists must be aware of it to ensure no malignant pathology is present and prevent further misdiagnosis. In this study, we present a case of EPT that we found incidentally during the histopathological examination of the specimen from the gallbladder in a patient who underwent cholecystectomy due to acute cholecystitis.

## 2 CASE HISTORY

A 40‐year‐old male was referred to the emergency department for acute pain in the right upper quadrant of the abdomen. The pain was constant and initiated hours before and intensified when he had dinner. He also reported fever, anorexia, nausea, and one involuntary vomiting episode containing only stomach contents. Besides intellectual disability and epilepsy, patient's medical history was unremarkable otherwise. He was on valproate (200 mg/D), risperidone (1 mg/BD), and clonazepam (1 mg/HS). He denied smoking and any recreational drug use. No allergies were reported. During the clinical examination, his vitals were slightly above normal ranges (blood pressure: 115/70 mmHg; heart rate: 108 bpm; respiratory rate: 19 breaths/min; temperature: 37.9°C; oxygen saturation: 99% without supplemental oxygen). The palpation of the abdomen revealed tenderness in the right upper quadrant and a positive Murphy sign with no rebound tenderness or guarding. The laboratory evaluation was within normal range except for a WBC count of 10,300, ESR of 32, and a 2+ CRP. Additionally, he underwent an ultrasonographic evaluation of the upper abdomen, reporting a thickened wall gallbladder containing multiple stones (measuring up to 8 × 10mm). The patient was scheduled for emergency laparoscopic cholecystectomy under the diagnosis of acute calculous cholecystitis in an about 12‐h period after initiation of the symptoms. During surgery, the gallbladder was found to be gangrenous. The patient had a complicated postoperative period due to surgical wound infections and was hospitalized for a week to receive intravenous antibiotics; however, he did not report any delayed complications or symptoms after discharge.

## 3 INVESTIGATIONS

**FIGURE 1:**
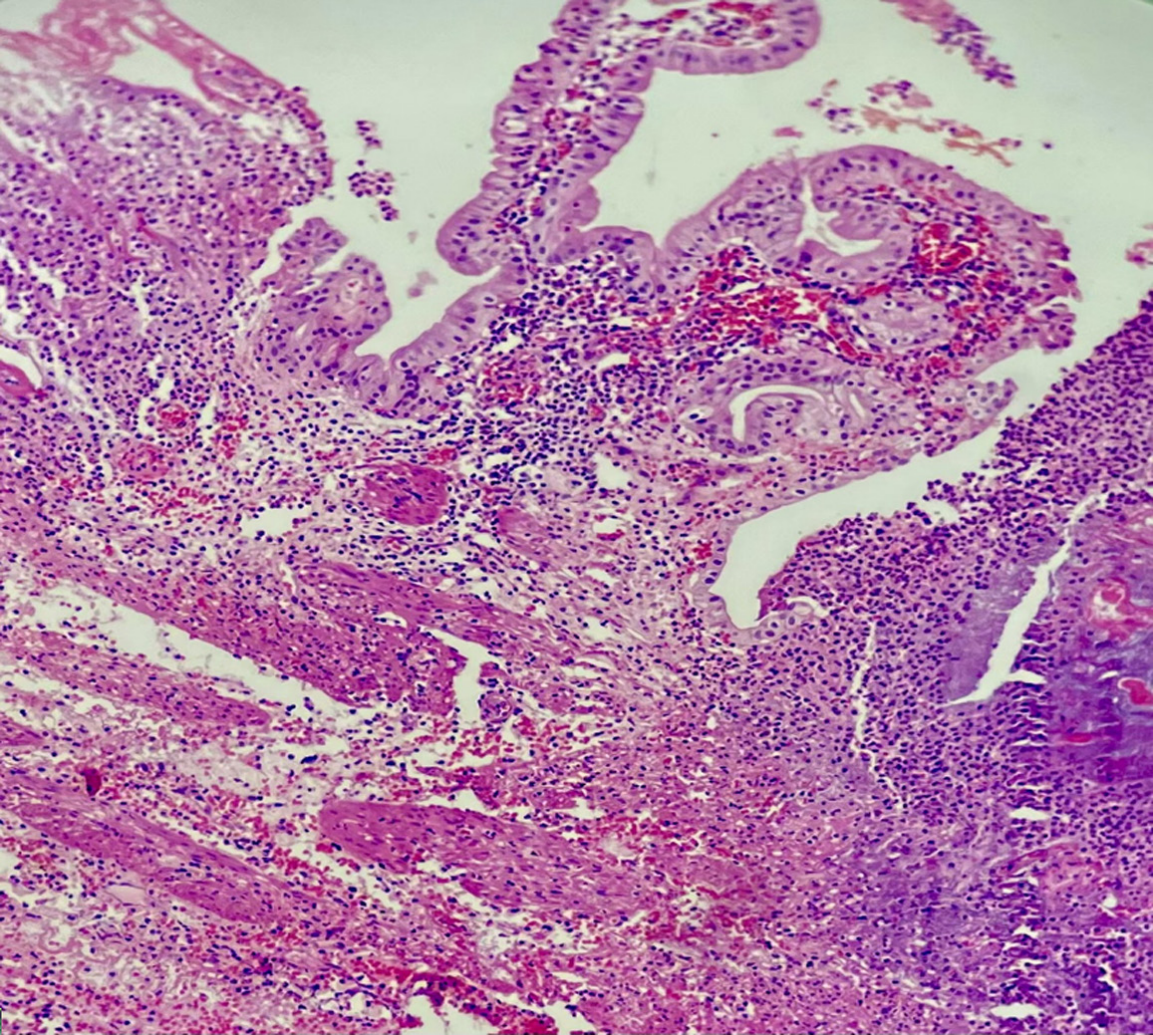
Acute gangrenous cholecystitis.

During pathologic examination, gallbladder measured 9 cm × 4 cm containing multiple small yellowish stones. The mucosa was green, and the maximum wall thickness was 2 mm. An area of wall thickening measuring 7 mm × 4 mm × 3 mm was noted in the neck region of the gallbladder. Microscopic examination revealed characteristics indicative of acute gangrenous cholecystitis (Figure 1). Regarding the wall thickening in the neck area described macroscopically, histopathology confirmed the presence of heterotopic pancreatic tissue composed of acini and ducts (exocrine pancreatic tissue only; no islets of Langerhans were detected). There was no evidence of malignancy or dysplasia in any of the sections examined.

## 4 DISCUSSION

Ectopic pancreatic tissue is a rare entity; however, its actual incidence in the gallbladder is unidentified, as a lack of clinical symptoms can complicate the diagnosis.^5^ It can occur anywhere, but the most common locations are the stomach (27.5%), duodenum (25.5%), colon (15.9%), jejunum, and spleen.^6^ By contrast, the presence of EPT in the gallbladder, lung mediastinum, liver, mesentery, and ileum is considered extraordinary.^2^, ^7^ EPT can be diagnosed in any age group, but most cases are between 40 and 60 years old. Albeit the male‐to‐female ratio of any other type of EPT is 3:1, there is a female predominance, specifically for EPT in the gallbladder, which may be due to the fact that cholecystitis‐related cholecystectomies are more prevalent in women.^2^, ^8^ The etiologies of EPT are still unclear, but three hypotheses about its origin have been proposed. The first theory, which is widely accepted, suggests that EPT separates from the primitive pancreas gland during the rotation of the gastrointestinal tract in the embryogenic period. The second theory suggests that the longitudinal growth of the intestine from the lateral budding of the rudimentary pancreatic tissue while penetrating the intestinal wall causes the irregular transportation of the pancreatic tissue.^9^, ^10^ The third theory supports that abnormalities in the notch signaling system can result in changes in different foregut endoderm tissue during embryogenesis.^11^ Jean Schultz first described the heterotropic pancreas in the 18th century; however, the first classification was made by Von Heinrich et al. in 1909, which was later modified by Fuentes in 1973 that consisted of four types^12^:

1. Type *one*: acini with ducts and islet‐like pancreatic gland (normal pancreatic tissue).
2. Type *two*: canalicular variant pancreatic duct.
3. Type *three*: exocrine pancreas with acinar tissue.
4. Type *four*: endocrine pancreas with cellular islets.

**FIGURE 2:**
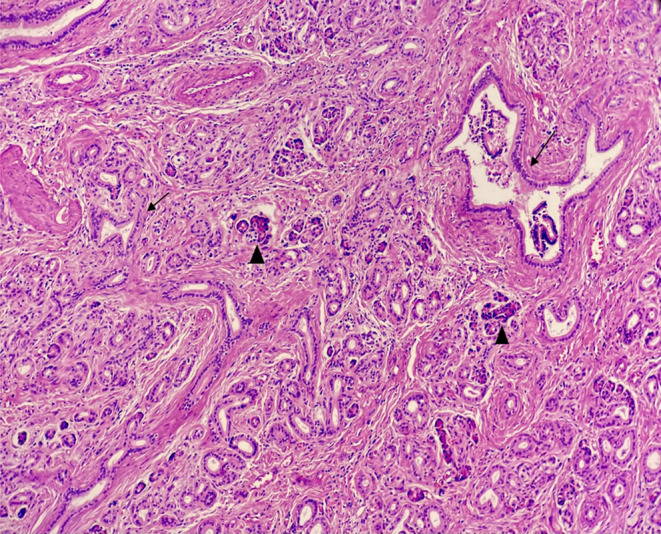
Heterotopic pancreatic islands consist of exocrine pancreas with acinar glands *arrow heads* and pancreatic ducts *arrows* (*×10*, *H&E*).

**FIGURE 3:**
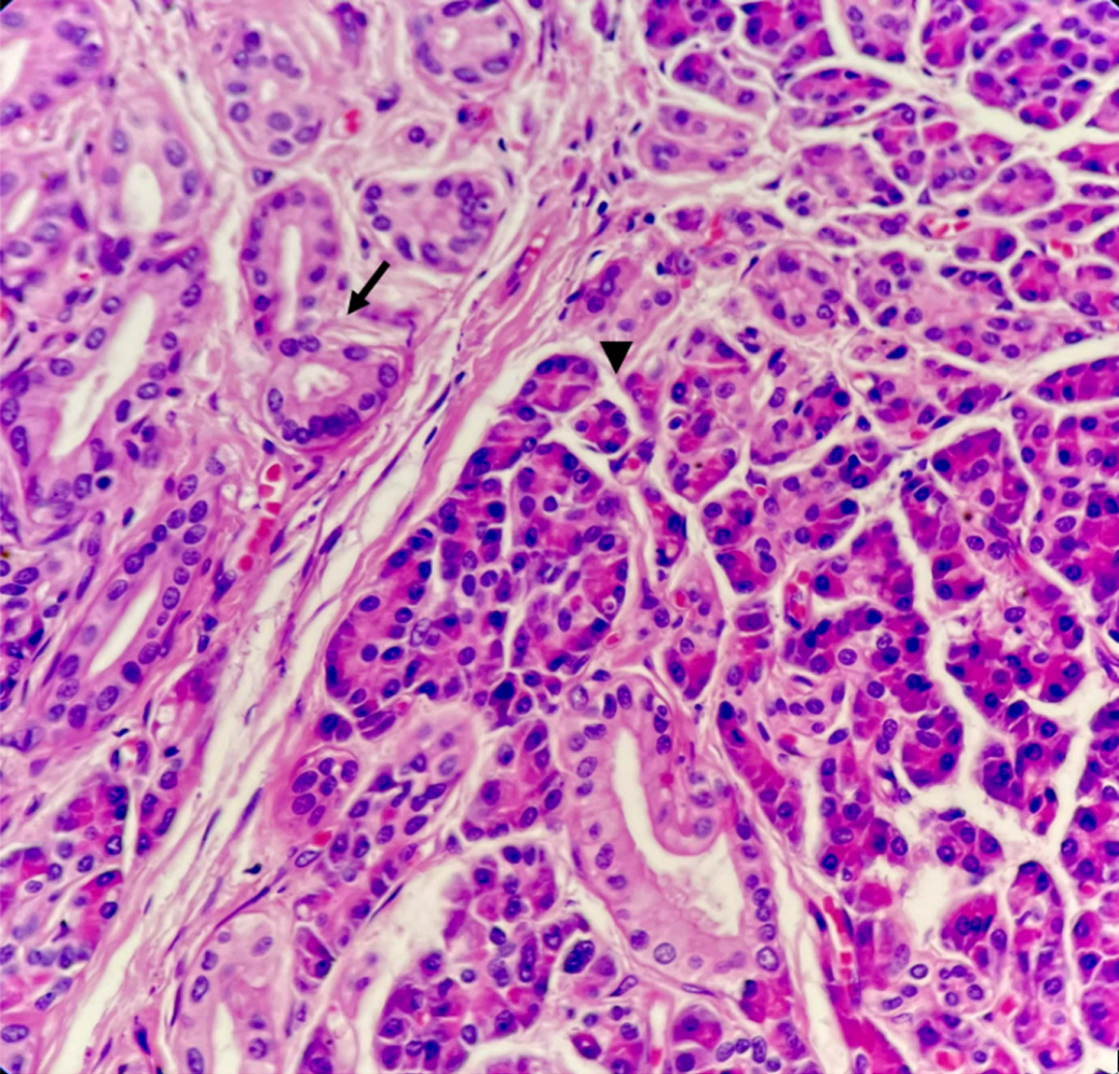
Heterotopic pancreatic islands consist of exocrine pancreas with acinar glands *arrow heads* and pancreatic ducts *arrows* (*×40*, *H&E*).

**FIGURE 4:**
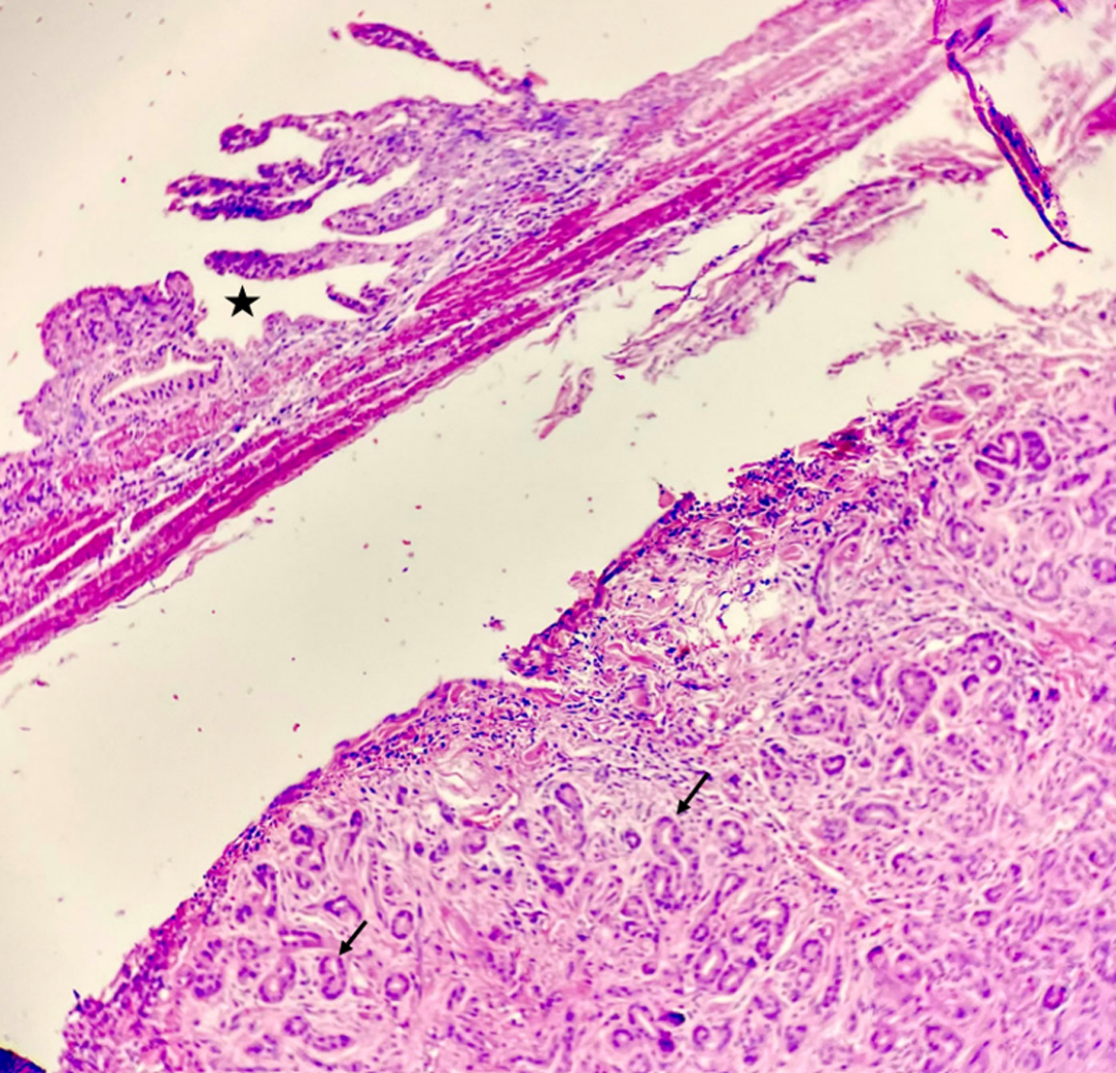
Small pancreatic ducts *arrows* with adjacent atrophic gallbladder mucosa *asterisk* (*×10*, *H&E*).

**FIGURE 5:**
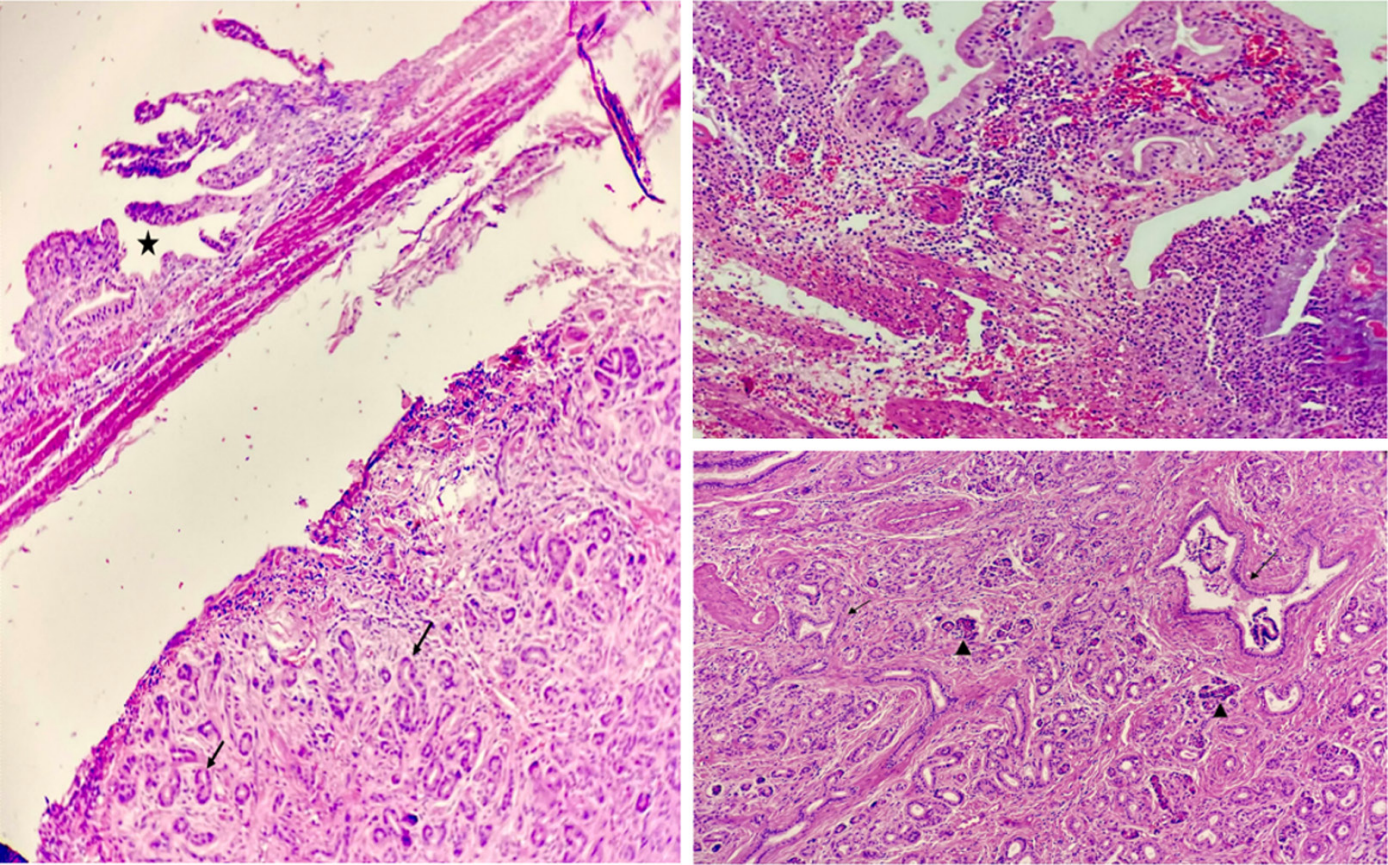
Histomorphology of gallbladder wall *asterisks* with an incidental erratic occurrence of heterotopic pancreatic islands *arrows and arrow heads* in an acute gangrenous cholecystectomy specimen *right upper corner*, masquerading malignancy in the initial sections of microscopic examination.

According to what is mentioned above, this case is compatible with type three (Figures 2, 3, 4, 5).

These ectopic pieces of tissue macroscopically can appear as an exophytic mass, similar to polypoid lesions, or as a nodule with a yellow‐colored appearance with sizes varying from a few millimeters to even 4 cm.^13^ Still, they are generally asymptomatic and only discovered histopathologically. However, in some cases, it can cause various nonspecific symptoms, depending on the location. Symptoms can include jaundice if obstructing bile ducts or biliary colic‐like symptoms (e.g., right upper quadrant pain, anorexia, nausea, and vomiting after meals); nevertheless, such symptoms presumably result from simultaneous lithiasic cholecystitis. Other conditions can be derived from EPT, such as cholelithiasis, acute or chronic cholecystitis, or carcinoma.^2^, ^10^

As Sato et al. reported that pancreatic enzymes (amylase and lipase) secreted from EPT in the gallbladder could impact its mucosa leading to gallbladder dysplasia and carcinoma. Therefore, cases with EPT in the gallbladder must undergo cholecystectomy as a definite treatment preventing any potential malignant transformation. Moreover, EPT can potentially cause the same pathologies as typical pancreatic tissue, which includes cysts, pseudocysts formation, abscess, and acute or chronic pancreatitis.^14^, ^15^ Table.1 provides a summarize literature review including the clinical presentation along with the histological characteristics of similar cases of EPT in the gallbladder.

**TABLE 1:** Recent literature review.

Reference	Presentation	Coexisting diagnosis*	Classification
Laslett et al.^16^	Chronic RUQ pain Postprandial nausea	Chronic cholecystitis	Type 1
Jansen van Rensburg et al.^17^	Chronic RUQ pain Fevers Vomiting	Chronic cholecystitis	Type 3
Serboiu et al.^18^ (3 cases)	Upper abdominal pain radiating to the back Nausea Vomiting	Acute cholecystitis	Type 1
	Chronic right Hypochondriac pain Nausea and vomiting	Chronic cholecystitis	Type 2
	RUQ abdominal pain Postprandial nausea and vomiting	Chronic Cholecystitis	Type 3
Al‐janabi et al.^19^	Chronic RUQ abdominal pain radiating to the back	Chronic cholecystitis	Type 1
Mishra et al.^20^	Chronic right hypochondriac pain Nausea and vomiting	Acute cholecystitis	Type 3

* The main underlying condition which led to cholecystectomy.

Diagnosing EPT in the gallbladder before and during an operation is impossible. Preoperative radiologic evaluation (ultrasound or computed tomography scan) usually cannot detect EPT in the gallbladder; neither this case did.^21^ Therefore, only a precise histopathologic examination can provide a definite diagnosis; consequently, it is essential for anatomical pathologists to be aware of this uncommon presentation of EPT in gallbladder to discriminate it from a masquerading malignancy.

To end with, the question that whether presence of EPT in gallbladder may have any role in the exacerbation/origination of an acute cholecystitis episode or not, remains unclear in this study. Hope future studies could better elucidate this association.

## AUTHOR CONTRIBUTIONS

**Nooshin Zaresharifi:** Conceptualization; data curation; investigation; methodology; project administration; resources; supervision; validation; writing – review and editing. **Anita Khalili:** Writing – original draft. **Behrad Eftekhari:** Writing – original draft. **Hojjat Layegh:** Conceptualization; supervision; writing – review and editing.

## FUNDING INFORMATION

None.

## CONFLICT OF INTEREST STATEMENT

None.

## ETHICS APPROVAL

Study was approved by the Research Ethics Committee of the Guilan University of Medical Sciences (reference number: IR.GUMS.REC.1402.241).

## CONSENT

Written informed consent was obtained from the patient to publish this report in accordance with the journal's patient consent policy.

## Data Availability

The data that support the findings of this study are available from the corresponding author upon reasonable request.
